# A Brain-Inspired Decision-Making Linear Neural Network and Its Application in Automatic Drive

**DOI:** 10.3390/s21030794

**Published:** 2021-01-25

**Authors:** Tianjun Sun, Zhenhai Gao, Fei Gao, Tianyao Zhang, Siyan Chen, Kehan Zhao

**Affiliations:** 1 State Key Laboratory of Automotive Simulation and Control, Jilin University, Changchun 130012, China; sun_tj@jlu.edu.cn (T.S.); gaozh@jlu.edu.cn (Z.G.); gaofei123284123@jlu.edu.cn (F.G.); tianyaz@jlu.edu.cn (T.Z.); 2 College of Automotive Engineering, Jilin University, Changchun 130012, China; 3 College of Materials Science and Engineering, Jilin University, Changchun 130012, China; zhaokh1619@mails.jlu.edu.cn

**Keywords:** brain-inspired decision-making, fuzzy classification, linear neural network, human-like automatic driving system

## Abstract

Brain-like intelligent decision-making is a prevailing trend in today’s world. However, inspired by bionics and computer science, the linear neural network has become one of the main means to realize human-like decision-making and control. This paper proposes a method for classifying drivers’ driving behaviors based on the fuzzy algorithm and establish a brain-inspired decision-making linear neural network. Firstly, different driver experimental data samples were obtained through the driving simulator. Then, an objective fuzzy classification algorithm was designed to distinguish different driving behaviors in terms of experimental data. In addition, a brain-inspired linear neural network was established to realize human-like decision-making and control. Finally, the accuracy of the proposed method was verified by training and testing. This study extracts the driving characteristics of drivers through driving simulator tests, which provides a driving behavior reference for the human-like decision-making of an intelligent vehicle.

## 1. Introduction

Human-like decision-making for an intelligent vehicle plays a paramount role in the development of brain-like intelligence [1]. However, existing studies mostly focus on rule-based control strategies, which depend on tedious rules and known scenarios [2,3]. Therefore, how an intelligent vehicle can explore or make decisions in unknown and complex environments that involve humans has become a hot topic at the intersection of bionic engineering and artificial intelligence.

In previous studies, researchers divided driving activities into three spaces (involving humans or driving machines): perception space, cognitive space, and physical space [4]. In the perception space, humans can obtain information from the surrounding environments through use of their sensory organs, such as eyes, ears, and hands. Homoplastically, an intelligent vehicle, will obtain the information from the states and surrounding environments through sensors, such as cameras and radars. In the cognitive space, humans make decisions using their minds, prior knowledge, and situational understanding. However, compared with human decision-making processes, an intelligent vehicle will perform actions through rule-based control strategies and environmental information. In the physical space, the traditional vehicle can be controlled by human limbs, but for an intelligent vehicle, it will be controlled by electrical signals and mechanical structures based on dynamics models. Thus, the human brain can accomplish learning and memory through cooperative work with different regions; thus, realize driving activities [5]. Therefore, inspired by human decision-making processes, brain-inspired mechanisms with computer technology deconstruction have become the latest international trends.

**Figure 1:**
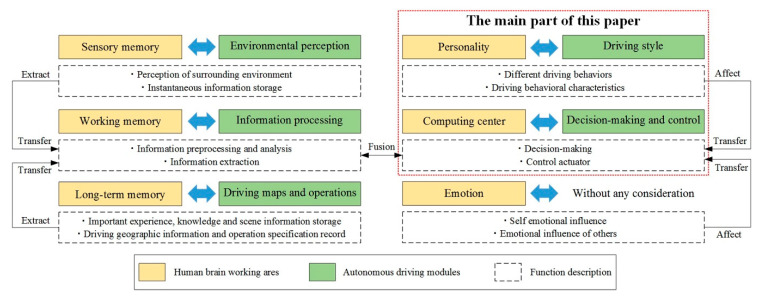
The corresponding relations between the human brain and an automatic driving system.

There are six functional areas in the human brain, which will be taken into consideration, sensory memory area, working memory area, long-term memory area, computing center area, emotion area, and personality area. In addition, through the understanding of brain-like intelligence applied to an intelligent vehicle, the corresponding relations between the working areas of the human brain and autonomous driving modules is shown in Figure 1.

Analyzing characteristic data during driver driving is a key piece of technology that solves decision-making problems in intelligent vehicles. However, when imitating the human-like decision-making process, characteristic data that involve moving vehicles become a bond between the driver and vehicle. Driving behavioral characteristics refer to drivers in their own factors, vehicle factors, environmental factors, and other factors, under the combined influence of the actual traffic situation, which presents the corresponding action and reflects the regularity of driver manipulation, to some degree, and can be understood as the driver’s intrinsic attributes. In early studies, for example, Abdul et al. [6] referenced the accelerator pedal and brake pressure as characteristic indexes for driving behavior, and established a driver’s behavior model based on the cerebellar model joint controller. By identifying the driver’s personality and subconscious, the internal connection between the driver’s behavioral characteristics and the cerebellar unconscious response were revealed. Liu et al. [7] built a driver action model based on the sequence of internal mental states, in which each state corresponded to a driver’s personalized control behavior pattern. By observing the current driver’s control behavior and comparing it with the established action model, the next action the driver takes can be predicted. Quintero et al. [8] established two models based on the back propagation (BP) neural network. One was used to classify drivers and identify real-time road conditions; the other was used to identify the state of the driver. Yingfan et al. [9] adopted different parameters and a combination for the driver’s braking intention recognition; the results showed the feasibility for a multi-parameter comprehensive analysis. Changfu et al. [10,11] combined the double hidden Markov model with a neural network model to discover the driver intention recognition under complex conditions, and driving behavior prediction through real driving simulator experiments. Zambrano-Martinez et al. [12] performed an experimental study of the traffic distribution in the city of Valencia, Spain, characterizing the different streets of the city in terms of vehicle load, with respect to travel time during rush hour traffic conditions. Zeng et al. [13] provided a new way to describe driving pedal behavior after decomposing it into a sequence of actions. By considering the vehicle and road information as the inputs and the pedal action as the output, an input-output hidden Markov model (IOHMM) was used to describe the pedal behavior. In summary, the conventional models lack consideration of driving styles, and a brain-inspired decision-making method has not been widely researched until recently.

This study aims to develop a mapping relationship between vehicle motion states and vehicle control outputs, and, at the same time, driving styles are taken into consideration. The main contributions of this study are as follows:

(1) The experimental data of different driving samples were obtained through the driving simulator and the characteristic parameters were extracted and analyzed.
(2) An objective fuzzy classification algorithm based on safety and efficiency was designed to distinguish different driving behaviors, in terms of experimental data.
(3) A brain-inspired linear neural network was established to realize human-like decision-making and control; accuracy was verified by training and testing.

The remainder of this paper is organized as follows: Section 2 is Related Works, which provides detailed information on our experimental methods, experimental data, and theoretical basis. Section 3 is Description and Analysis of Characteristic Data, which provides the analysis of characteristic data with driving simulator tests. Section 4 is the Design of Fuzzy Classification Algorithm, which provides the design methodology and algorithmic architecture. Section 5 is the Linear Neural Network for Brain-Inspired Decision-Making, which provides the algorithm construction process and simulation results. Section 6 is Conclusions and Future Works, which provides the conclusions of our study, and conceptions we should ensue further. The final section is Discussions, which provides some current deficiencies and limitations.

## 2. Related Works

**Figure 2:**
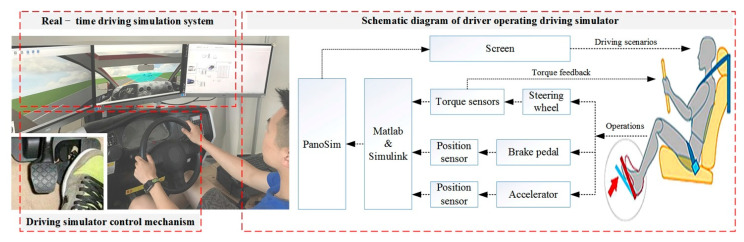
The process of experimental data collection based on the driving simulator.

In order to reduce the cost and consumption of resources (e.g., humans, material, and financial resources generated by the real vehicle test), the driving simulator experiment not only saved costs, but also improved safety and controllability. We used the driving simulator with PanoSim to establish simulated driving scenarios, which provided experimental data through the modular program with real-time acquisition. According to the driving scenarios presented by PanoSim, and the current driving condition of the vehicle, the state of the vehicle could be changed by the driver, who controlled the steering wheel, accelerator pedal, or brake pedal. Then, a Simulink model was prepared for receiving the signals from the angle sensor, torque sensor, and speed sensor. In addition, these data were recorded through the module of “To Workspace” based on MATLAB. Finally, the vehicle motion states and driving environment were presented on screen, as shown in Figure 2.

In our related works, we recruited 100 driving samples that included different genders, ages, occupations, and driving years, from schools and society. However, based on these driver samples, the significant differences emerged through experiments. At first, in order to eliminate the strangeness, we instructed participants to try to use the driving simulator because most of participants were unfamiliar with the machine. Then, after two rounds of testing, which took about 20 min, they did a formal test, which took about 10 min. In a formal test, the velocity, acceleration/deceleration, relative distance, acceleration pedal opening degree, and brake master cylinder pressure are collected based on the same driving conditions with different driver samples.

**Figure 3:**
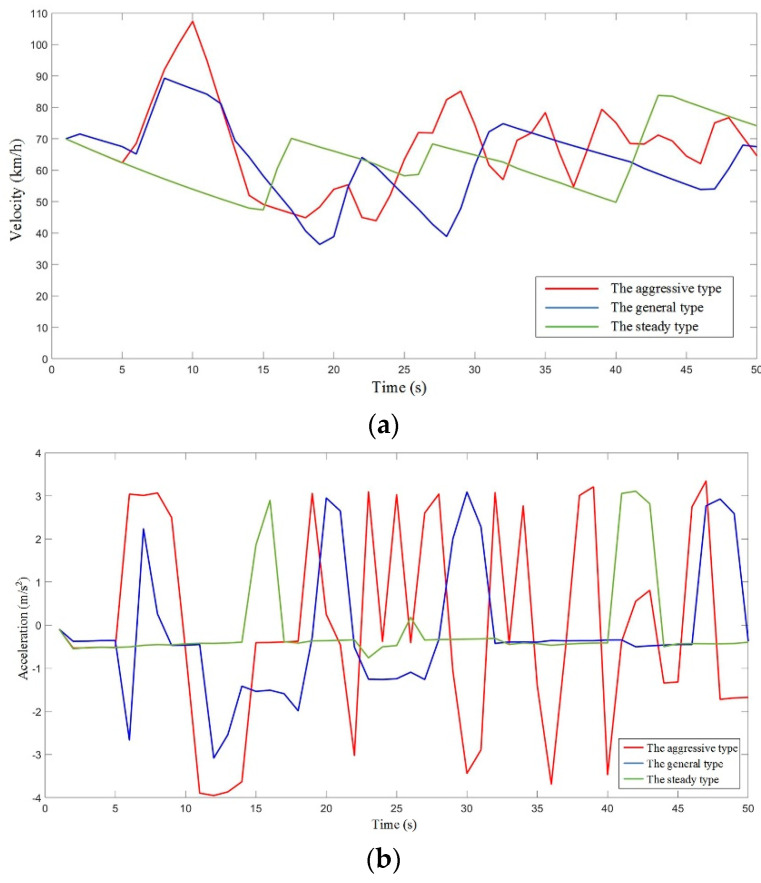

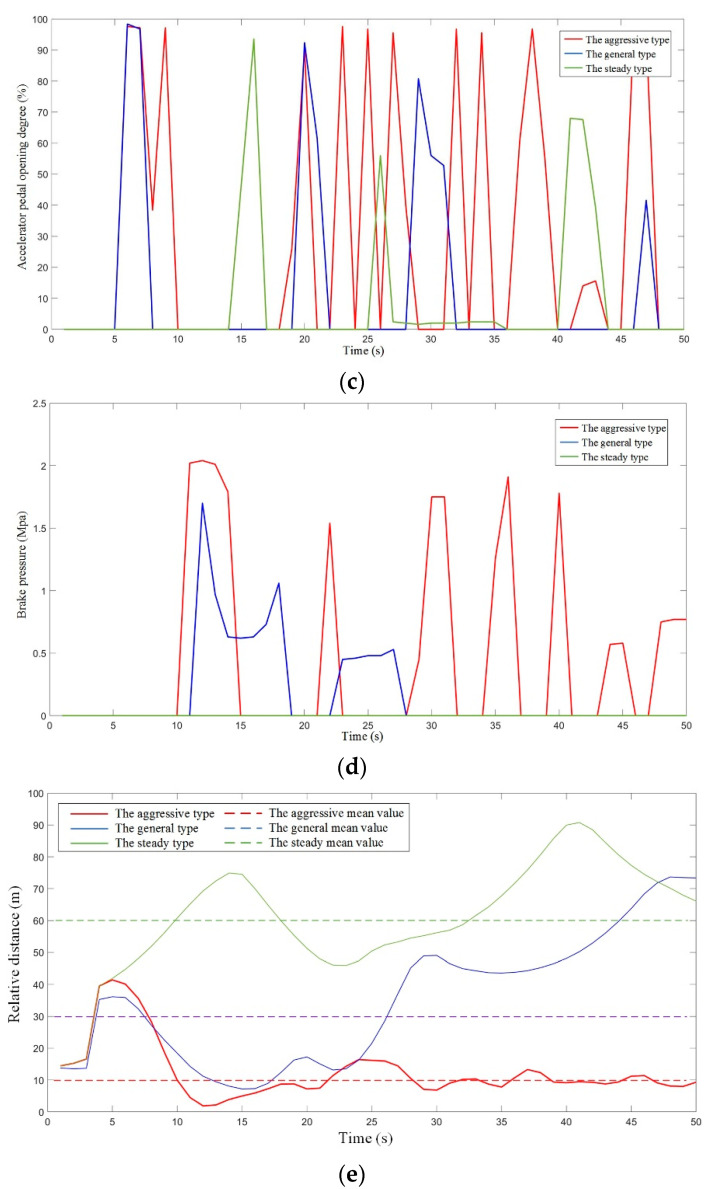
The experimental results for the curves after fitting with the three driving types: (**a**) the velocity for three driving types; (**b**) the acceleration for the three driving types; (**c**) the accelerator pedal degree for the three driving types; (**d**) the brake pressure for the three driving types; and (**e**) the relative distance for the three driving types.

Considering our goal in this study, we mainly focused on car-following driving conditions that took place on a straight road with different driver samples. Here, we compare the mean square error values among 100 driver samples, because the mean square error is usually represented as the degree of dispersion of a dataset. A large mean square error means that most values in the dataset have a large difference from their mean value, while a small mean square error means that most values in the dataset are close to the population mean value. Furthermore, we calculate the mean value of the square error mean in order to distinguish different types of driver samples. In this way, it is not appropriate to use all of data in the following algorithm design. Thus, if we divide these driver samples into three parts, based on the mean square error, then we can fit out three curves, representing an aggressive driver, a general driver, and a steady driver, without consideration of gender. The initial 100 driver samples were prepared for the different styles; however, these three curves, after fitting for the three driving types, were prepared for typicality. In this way, we can draw conclusions through these curves, which are shown in Figure 3.

We chose 100 driver samples for the experimental data collection, as mentioned previously. From the samples, we chose three who were professional drivers (and who had 10 years of driving experience) to be passenger evaluators. These three drivers were prepared for the occupant evaluations because of their specialties. Moreover, considering the general passenger vehicle, there were three seats available—except for the driver’s seat. The questionnaire for occupant evaluation is shown in Table 1.

Using the above method, we can obtain three passenger evaluator results for each driver sample. If the results of the three evaluators are consistent, the evaluation result should be regarded as the final passenger evaluator result. If one is inconsistent with the others, the majority of the evaluation results will be regarded as the final evaluation results of the passenger evaluators. If the results of the three evaluations are not consistent, the experimental test will be conducted again.

Our research group also conducted experimental studies in the early stages. For example, Tianjun et al. [14] designed a decision-making method for vehicle longitudinal automatic driving based on reinforcing Q-learning, providing a driving behavior reference for the human-like vehicle control of an intelligent vehicle. Moreover, they designed an automatic braking system, considering driver-braking characteristics, providing a dataset for different driver-braking behaviors [15]. Therefore, we have a research basis for the conducted experiments and design Algorithms based on driver classification.

## 3. Description and Analysis of Characteristics Data

In our studies, 100 driver samples were prepared for experimental testing by a driving simulator. However, not all of the data were representative. In order to provide a better illustration on the different types of driving behaviors, gender was simultaneously taken into consideration. The traditional classification method based on characteristic mean value is regarded as a relatively crude method, which cannot be used as a judgment basis for detailed divisions of driving styles. Therefore, in this part, the driver samples were divided into six driving types, according to the previous classification method, by considering gender; then, through the analysis of data for different driving types after fitting, some key parameters were calculated to provide a better illustration for different types of driving behaviors.

**Figure 4:**
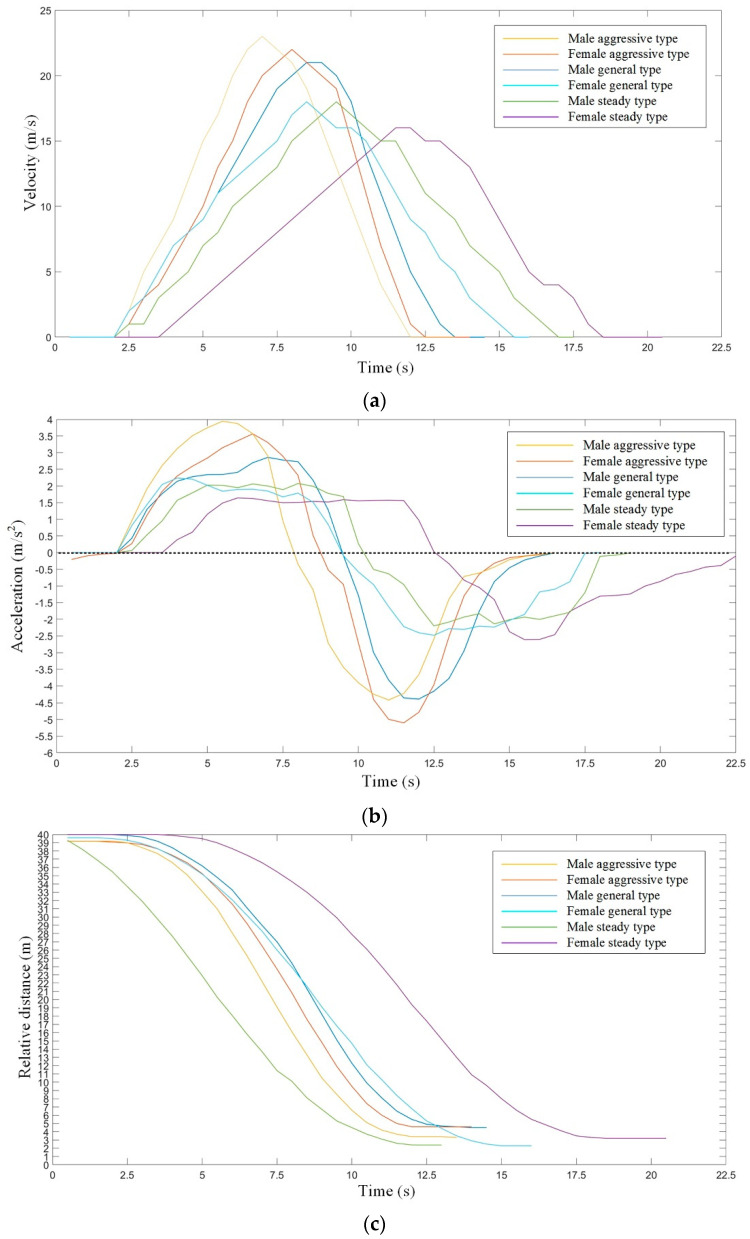
The experimental results for different driving types with different genders based on car-following: (**a**) the velocity for six driving types; (**b**) the acceleration for six driving types; and (**c**) the relative distance for six driving types.

Car-following, which is the most common driving condition, played an important role in both the simulated driving test and real driving test. There are three key parameters in the process of car-following: velocity (*V_current_*), longitudinal acceleration (*a*), and relative distance (*D_relative_*) [16,17]. The experimental results are shown in Figure 4.

Figure 4a shows that aggressive type and male general type tend to speed up quickly, while the female general type and steady type tend to speed up slowly. At the same time, during the deceleration process, the aggressive type and male general type tend to slow down faster, while the female general type and steady type tend to slow down slower. Figure 4b shows that the acceleration range of the aggressive type and male general type are large during the car-following process, which can lead to an obvious feeling of pushback when accelerating, and an obvious feeling of “nod-head” when decelerating. However, the female general type and steady type have a narrow acceleration range. Figure 4c shows that the relative distance after braking for the male aggressive type and female steady type are about 3.9 m, which, for the female aggressive type and male general type are about 5.1 m, and for the female general type and male steady type are about 2.3 m. An obvious difference existed in different genders with the same driving types.

Thus, in order to reflect the different driving behaviors in the car-following process, by analyzing the above data, further information can be obtained from these results, such as the maximum of longitudinal acceleration (*a_max_*), the minimum of longitudinal deceleration (*a_min_*), the average absolute value of acceleration ($\bar{a}$), the absolute variance of acceleration ($\sigma_{a}$), the expected distance between two vehicles (*d_safe_*), and the safety distance after braking (*d_emergency_*), as shown in Table 2.

In Table 2, $\bar{a}$ represents the average absolute value of acceleration, the calculation method is shown in Equation (1); $\sigma_{a}$ represents the absolute variance of acceleration; the calculation method is shown in Equation (2); *d_safe_* represents the expected distance between two vehicles, which can be obtained from the data in Figure 3e; *d_emergency_* represents the safety distance after braking, which can be obtained from the data in Figure 4c.

(1) $$\bar{a}=(|a_{1}|+|a_{2}|+\cdots +|a_{n}|)/n$$

(2) $$\begin{array}{c} \sigma_{a}=|[{\left(a_{1}-M\right)}^{2}+{\left(a_{2}-M\right)}^{2}+\cdots +{\left(a_{n}-M\right)}^{2}]/n|,\\ \;M=(|a_{1}|+|a_{2}|+\cdots +|a_{n}|)/n \end{array}$$

## 4. Design of Fuzzy Classification Algorithm

According to the fuzzy theory [18,19], driving behavioral characteristics are not an absolute concept, or “black or white” in the traditional sense, but they have a mutual connection with each other. In order to achieve a more accurate classification through the experimental data, we established two key indicators corresponding to characteristic parameters: safety indicator and efficiency indicator.

In this study, we assume that the driving behaviors are aggressive and steady for the following reasons: on the one hand, we described the driving behaviors for different drivers by mainly focusing on the average driving speed; thus, it was easy to divide drivers into two groups depending on average speed. On the other hand, in terms of the probability calculation, it is convenient for the calculation when only considering two types of driving behaviors. According to the naive Bayesian model, the problem with driving behavioral classification is transformed into a probabilistic problem. There are two types of driver samples: one is an aggressive driver, denoted as *C*_1_, the other is a steady driver, denoted as *C*_2_. Thus, if the driver sample is denoted as *x*, then the probability represents the driver sample (*x*) belongs to an aggressive driver (*C*_1_) can be expressed as *P*(*x*|*C*_1_)*P*(*C*_1_). However, the probability that the driver sample (*x*) belongs to a steady driver (*C*_2_) can be expressed as *P*(*x*|*C*_2_)*P*(*C*_2_). In this way, the total probability can be calculated in Equation (3):

(3) $$P(x|C_{1})P(C_{1})+P(x|C_{2})P(C_{2})=1$$

After the transformation, the probability that the driver sample (*x*) belongs to an aggressive driver (*C*_1_) can be expressed as Equation (4):

(4) $$P(x|C_{1})P(C_{1})=\frac{1}{1+\frac{P(x|C_{2})P(C_{2})}{P(x|C_{1})P(C_{1})}}$$

If $z=\ln\frac{P(x|C_{1})P(C_{1})}{P(x|C_{2})P(C_{2})}$ and $P(x|C_{1})P(C_{1})=\sigma (z)$, then the Equation (4) will be rewritten as Equation (5):

(5) $$\sigma (z)=\frac{1}{1+e^{-z}}$$

**Figure 5:**
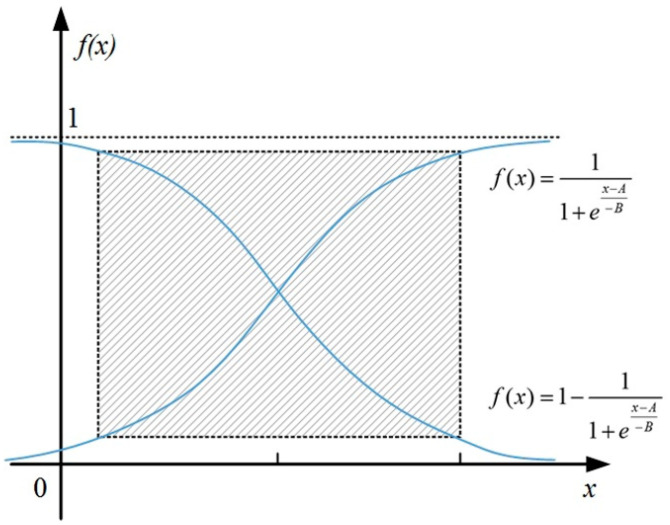
The traditional sigmoid function.

The traditional sigmoid function is shown in Equation (6) and Figure 5.

(6) $$f(x)=\frac{1}{1+e^{\frac{x-A}{-B}}}\;\mathrm{or}\;f(x)=1-\frac{1}{1+e^{\frac{x-A}{-B}}}$$

where *x* represents the variable, *A* represents the value of *x* when *f*(*x*) = 0.5, *B* represents the trend of gain, which can be calculated with detailed information.

The sigmoid function, which operates on the neurons of the neural network, maps the input of the neuron to the output. It can be imagined as the firing rate of a neuron, where the high slope in the middle is the sensitive area of the neuron, and the gentle slope on both sides is the inhibitory area of the neuron. Mathematically, the sigmoid function has a large signal gain for the central region and a small signal gain for the two sides, which has a good effect on the signal feature space mapping. From the perspective of neuroscience, the central region is similar to the excited state of neurons, and the bilateral region is similar to the inhibited state of neurons. Therefore, in terms of neural network learning, the key features can be pushed to the central region, and the non-key features to the bilateral regions.

**Figure 6:**
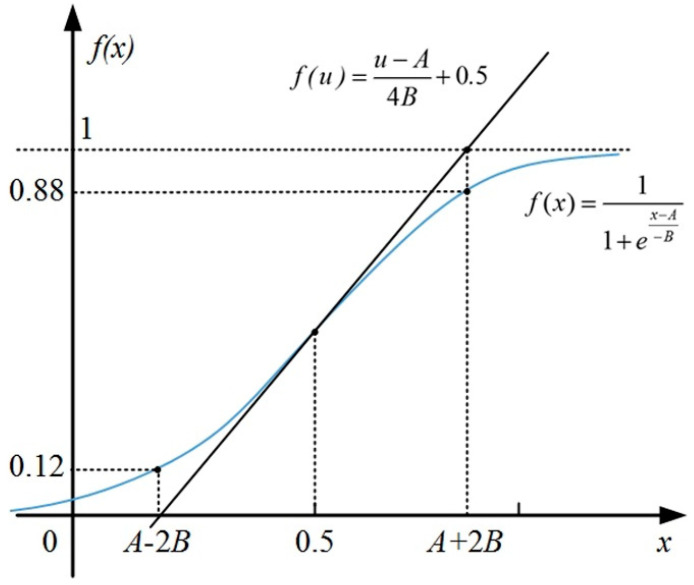
The tangential equation for sigmoid function.

Furthermore, the tangential equation *f*(*u*) at the point of (*A*, 0.5) can be calculated in Equation (7) and Figure 6.

(7) $$f(u)=\frac{u-A}{4B}+0.5$$

As shown in Figure 6, we can see that the tangent intersects with *f*(*x*) = 0 and *f*(*x*) = 1. Thus, it may produce two points of intersection, the x-coordinates are *A − 2B* and *A + 2B*. Moreover, combined with Equation (6), if *x = A* *− 2B*, then *f*(*x*) = 0.12; if *x = A + 2B*, then *f*(*x*) = 0.88. The detailed calculation process is shown as follows:

If $f(x)=\frac{1}{1+e^{\frac{x-A}{-B}}}$ and *x = A* − *2B*,

Then $f(x)=\frac{1}{1+e^{\frac{A-2B-A}{-B}}}=\frac{1}{1+e^{\frac{-2B}{-B}}}=\frac{1}{1+e^{2}}\approx \frac{1}{1+7.3891}\approx 0.12$;

Else if $f(x)=\frac{1}{1+e^{\frac{x-A}{-B}}}$ and *x* = *A* + *2B*,

Then $f(x)=\frac{1}{1+e^{\frac{A+2B-A}{-B}}}=\frac{1}{1+e^{\frac{2B}{-B}}}=\frac{1}{1+e^{-2}}\approx \frac{1}{1+0.1353}\approx 0.88$.

The fuzzy classification algorithm for driving behavior is the premise of human-like brain-inspired decision-making. In the process of designing the fuzzy classification algorithm, according to the above theoretical basis and previous studies [20,21], safety and efficiency are taken into consideration, and characteristic data will be used for analysis.

### 4.1. Safety Indicator

**Figure 7:**
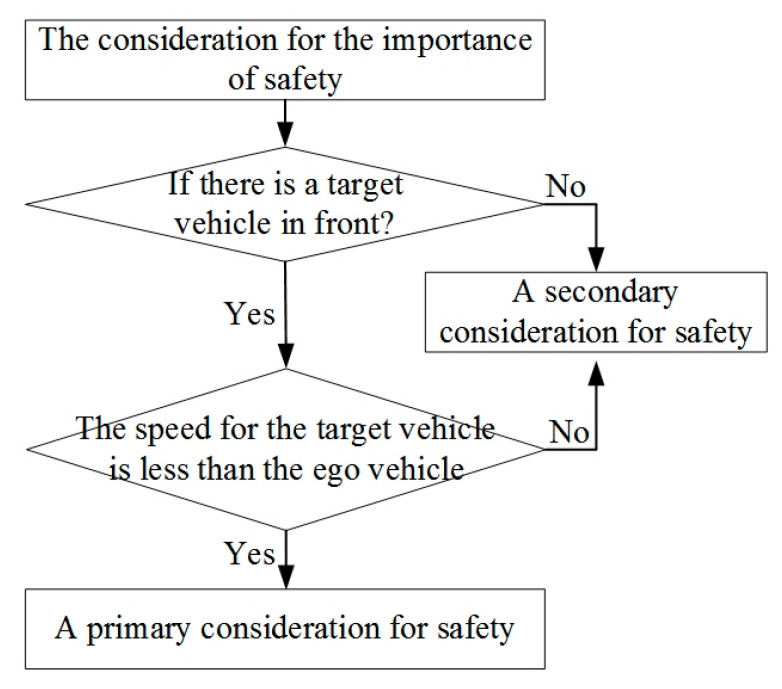
The consideration for the importance of driving safety indicators.

As shown in Figure 7, when a driver follows a vehicle, his brain mainly considers whether there are obstacles ahead. If there is a target vehicle in front, and its speed is less than the ego vehicle, then it becomes a typical condition, which needs to consider driving safety. With the relative distance gradually decreasing, once the distance is less than the safety distance, the vehicle will be in a relatively dangerous driving state, and a collision accident may occur. If there is no target vehicle in front, or if its speed is faster than the ego vehicle, the relative distance will be larger and larger, so there will be no collision accident by default, and the consideration of safety can be put in second place, while the consideration of efficiency can be put in first place.

**Figure 8:**
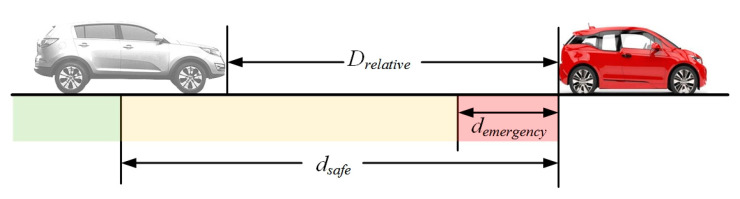
The relative distance division in the process of car-following.

According to the theory of the rigid body collision and vehicle kinematics, the relative distance involved in the process of car-following can be divided based on the degree of danger, as shown in Figure 8. The green part (*D_relative_* > *d_safe_*) means that when the actual relative distance between two vehicles is larger than the safety distance, then the ego vehicle is in a safe state. The yellow part (*d_emergency_* < *D_relative_* < *d_safe_*) means that the actual relative distance between two vehicles is smaller than the safety distance, but if the ego vehicle brakes, then the collision will not happen. The red part (*D_relative_* < *d_emergency_*) means that the actual relative distance between two vehicles is smaller than the danger distance, and then the collision will happen. In the above process, the safety that we mainly considered is reflected in the yellow part—that is, the corresponding condition can be described as the target vehicle being stationary or braking suddenly.

Based on the above analysis, when the actual relative distance between two vehicles is in the green part, the driving safety is the best. When the actual relative distance between two vehicles is in the red part, the driving safety is the worst and there will be a collision. Therefore, *d_safe_* and *d_emergency_* are selected as the high-gain and low-gain boundaries of the sigmoid function. At the same time, by considering driving safety, we further calculate the mean value of *d_safe_* and *d_emergency_* with general drivers, including a male driving type and a female driving type. Combined with Equation (6), the value of *A*_1_ and *B*_1_ can be calculated and the membership function for safety indicator (*f*(*w*)*_s_*) will be represented as Equation (8):

(8) $$f{\left(w\right)}_{s}=\frac{1}{1+e^{\frac{x-A_{1}}{-B_{1}}}},\;\left\{ \begin{array}{l} A_{1}=(d_{safe}+d_{emergency})/2\\ B_{1}=(d_{safe}-d_{emergency})/4 \end{array}\right.,\;\left\{ \begin{array}{l} d_{safe}=A_{1}+2B_{1}\\ d_{emergency}=A_{1}-2B_{1} \end{array}\right.$$

However, the detailed calculation process is shown as follows:

$$\begin{array}{l} d_{safe}=(d_{safe\_male}+d_{safe\_female})/2=(30+30)/2=30\\ d_{emergency}=(d_{emergency\_male}+d_{emergency\_female})/2=(4.5+2.3)/2=3.4\approx 3 \end{array}$$

Then, the value of *A*_1_ and *B*_1_ can be calculated based on above values, which are shown as follows:

$$\left\{ \begin{array}{l} A_{1}=(d_{safe}+d_{emergency})/2=(30+3)/2=16.5\\ B_{1}=(d_{safe}-d_{emergency})/4=(30-3)/4=6.75 \end{array}\right.$$

In this way, Equation (8) can be represented as Equation (9):

(9) $$f{\left(w\right)}_{s}=\frac{1}{1+e^{\frac{x-16.5}{-6.75}}}$$

where *w* represents the relative distance.

Moreover, the larger the value of this function, the higher the safety will be and the steadier the driving behavior will be. The smaller the value of this function, the worse the safety will be and the more aggressive the driving behavior will be. However, the value of *f*(*w*)*_s_* is continuous during the experimental calculation, so the mean value needs to be further calculated to represent the average driving safety degree in the current driving condition, which is calculated in Equation (10):

(10) $$\bar{f}{\left(w\right)}_{s}=[f{\left(w\right)}_{s_{1}}+f{\left(w\right)}_{s_{2}}+\cdots +f{\left(w\right)}_{s_{n}}]/n$$

### 4.2. Efficiency Indicator

Drivers are always expected to arrive at the destination as fast as possible when the human brain is not stimulated by a sense of danger, which is represented as the driving efficiency. In an urban traffic environment, when the traffic light changes from red to green, it means that all waiting vehicles can start to pass. At this time, the driver will accelerate as fast as possible to complete the vehicle starting control. However, the actual situation is not so. During the starting process, the vehicle will be restricted by the traffic environment, such as speed limit or a narrow road in front. It will also be affected by the dynamic characteristics of the vehicle itself. Finally, the driver cannot accelerate without considering the limits and comprehensive effects of the traffic environment.

Therefore, the expected velocity of the ego vehicle will be represented as another characteristic value for the proposed sigmoid function. In addition, the minimum of the expected velocity can be obtained by the minimum deceleration, based on the time headway (*THW*). On the contrary, the maximum expected velocity could be obtained by the maximum acceleration, based on time headway (*THW*). At last, the minimum value and the maximum value of the expected velocity are selected as the low-gain and high-gain boundaries of the sigmoid function. The membership function for the efficiency indicator (*f*(*o*)*_e_*) will be represented as Equation (11):

(11) $$f{\left(o\right)}_{e}=\frac{1}{1+e^{\frac{o-A_{1}}{-B_{1}}}},\;\left\{ \begin{array}{l} A_{1}=v_{0}+\frac{THW}{2}(|a_{\max}|-|a_{\min}|)\\ B_{1}=\frac{THW}{4}(|a_{\max}|+|a_{\min}|) \end{array}\right.,\;THW=D_{relative}/V_{current}$$

where *o* represents the velocity; *v_0_* represents the average velocity of general type. The driving efficiency becomes the worst if the value is set as the minimum acceleration, while it becomes the best if the value is set as the maximum acceleration.

In this way, Equation (11) can be represented as Equation (12):

(12) $$f{\left(o\right)}_{e}=\frac{1}{1+e^{\frac{o-[v_{0}+3.67THW]}{0.375THW}}}$$

Moreover, the larger the value of this function, the better the efficiency is, which means the more aggressive the driving behavior will be. The smaller the value of this function, the worse the efficiency is, which means the steadier the driving behavior will be. However, the function *f*(*o*)*_e_* is continuous during the experimental calculation, so the mean value needs to be further calculated to represent the average driving efficiency degree in the current driving condition, which is calculated in Equation (13):

(13) $$\bar{f}{\left(o\right)}_{e}=[f{\left(o\right)}_{e_{1}}+f{\left(o\right)}_{e_{2}}+\cdots +f{\left(o\right)}_{e_{n}}]/n$$

### 4.3. Fuzzy Classification Algorithm

**Figure 9:**
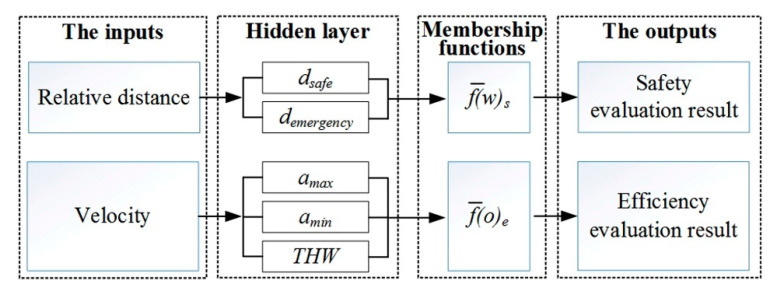
The architecture scheme of fuzzy classification Algorithm.

The fuzzy classification process is shown in Figure 9. The input of the fuzzy model is the relative distance and velocity, and the output is an evaluation result with safety and efficiency. Among them, there is a “Hidden layer” section between “The inputs” section and “Membership functions” section, which provides two options: if the input is the relative distance, then the hidden layer contains *d_safe_* and *d_emergency_*; if the input is the velocity, then the hidden layer contains *a_max_*, *a_min_* and *THW*.

In addition, the difference value between the safety evaluation result and efficiency evaluation result is represented as $\sigma_{f_{i}}$, which can be calculated in Equation (14):

(14) $$\sigma_{f_{i}}={\bar{f}}_{i}{\left(w\right)}_{s}-{\bar{f}}_{i}{\left(o\right)}_{e},\;i=1,2,\cdots ,5,6$$

Then, the mean value of $\sigma_{f_{i}}$ for six driving types will be calculated in Equation (15):

(15) $${\bar{\sigma }}_{f}=\sum_{i=1}^{6}\left[{\bar{f}}_{i}{\left(w\right)}_{s}-{\bar{f}}_{i}{\left(o\right)}_{e}\right]/6$$

We can draw some conclusions that the larger the value of $\sigma_{f_{i}}$, the more obvious the driving behavior will be, which can be boiled down to aggressive type or steady type. On the contrary, the smaller the value of $\sigma_{f_{i}}$, the less obvious the driving behavior will be, which can be boiled down to general type.

However, large or small are ambiguous concepts. We need to make a further classification by the comparison between $\sigma_{f_{i}}$ and ${\bar{\sigma }}_{f}$: if $\sigma_{f_{i}}<{\bar{\sigma }}_{f}$, then the classification result is general type; if $\sigma_{f_{i}}>{\bar{\sigma }}_{f}$, then the classification result is aggressive type or steady type. Thus, we have to distinguish between aggressive type and steady type by the weighting method.

We assume that the weighting coefficient *α* belongs to ${\bar{f}}_{i}{\left(w\right)}_{s}$ and the weighting coefficient *β* belongs to ${\bar{f}}_{i}{\left(o\right)}_{e}$, and *α + β =* 1. According to whether there is a target vehicle in front and the movement of the target vehicle, the value of the weighting coefficients are different:

(1) There is a target vehicle in front or the target vehicle is in an emergency driving condition. The weighted value *R^+^* based on composite indicators is calculated in Equation (16):
  (16) $$R^{+}=\alpha {\bar{f}}_{i}{\left(w\right)}_{s}+\beta {\bar{f}}_{i}{\left(o\right)}_{e},\;\left\{ \begin{array}{l} \alpha =0.6\\ \beta =0.4 \end{array}\right.,$$

Among them, we assume that *α > β* according to the past experience. If 0.12 *< R^+^ <* 0.5, then the driving behavior belongs to aggressive type; if 0.5 *< R^+^ <* 0.88, then the driving behavior belongs to steady type.

(2) There is no target vehicle in front. The weighted value *R^−^* based on composite indicators is calculated as shown in Equation (17):
  (17) $$R^{-}=\alpha {\bar{f}}_{i}{\left(w\right)}_{s}+\beta {\bar{f}}_{i}{\left(o\right)}_{e},\;\left\{ \begin{array}{l} \alpha =0.4\\ \beta =0.6 \end{array}\right.$$

Among them, we assume that *α < β* according to the past experience. If 0.12 *< R^−^ <* 0.5, then the driving behavior belongs to steady type; if 0.5 *< R^-^ <* 0.88, then the driving behavior belongs to aggressive type.

**Figure 10:**
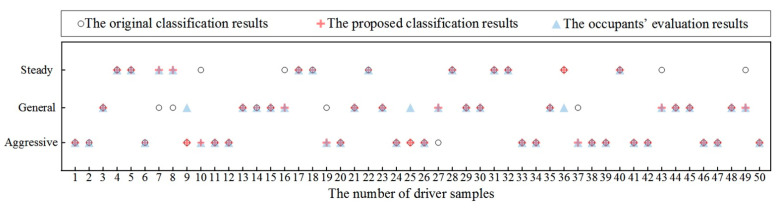
The experimental verification results based on 50 driver samples.

Thus, compared with the conventional classification methods, the proposed fuzzy algorithm based on intuitive parameters (such as relative distance and velocity) imitates the brain-like classification process. Finally, we carried out experimental verification on 50 driver samples and the results are shown in Figure 10.

There are two points to illustrate the experimental verification results:

(1) If the original classification result is the same as the proposed classification result, then the final result for driving behavior can be obtained.
(2) If the original classification result is different from the proposed classification result, then we will tend to the occupant’s evaluation result. In addition, if the proposed classification result is the same as the occupant’s evaluation result, then the final result for the driving behavior can be obtained. However, if the original classification result is the same as the occupant’s evaluation result, then we will check our algorithm and run the test again.

In this way, the consistency between the original classification result and the occupant’s evaluation result is 78%. Homoplastically, the consistency between the proposed classification result and the occupant’s evaluation result is 94%. Thus, the proposed human-like fuzzy algorithm improves the accuracy of classification, which also provides a reference for brain-inspired linear neural network decision-making.

## 5. Linear Neural Network for Brain-Inspired Decision-Making

Linear neural network consists of a single or multiple linear neurons. The differences between linear neural network and perceptron are as follows: the transfer function of each linear neuron is a linear function, so the output is an interval value (arbitrary value); the transfer function of the perceptron is a symbolic function, so the output is −1 or 1. The learning rules of the linear neural network adopt the least mean square (LMS) algorithm. The basic idea of this learning rule is to find the best weight and threshold to minimize the mean square error of output for each neuron. The main application fields of the linear neural network are function-fitting, approximation, prediction, pattern recognition, and so on [22].

**Figure 11:**
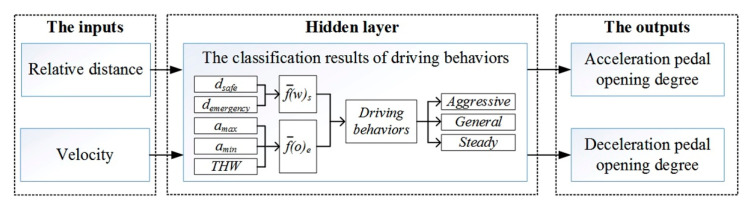
The architecture of linear neural network based on fuzzy classification Algorithm.

By reviewing the cognitive decision-making mechanism of human beings, the brain-inspired mechanism is mainly reflected in “end-to-end” inputs and outputs of neural networks. However, if a controller can provide the pedal opening degree directly through the relative distance and velocity, then it will become a human-like controller, which is similar to brain-like decision-making. Multiple outputs can be generated if the network contains multiple neuronal nodes. This linear neural network is called the Madaline network. Figure 11 shows the process of establishing a Madaline network based on linear neural network.

The shining point of the linear neural network is its LMS learning Algorithm, also called “Δ rules”. The LMS Algorithm can only train a single-layer network, but this will not have a great impact on its function. The objective of the linear neural network learning is to find the appropriate weight (*ω*) to minimize the value of the mean square error (*mse*). In order to find the minimum value, we need to use the method of derivative: just use *mse* to find the partial derivative, with respect to *ω*, and then set the partial derivative equal to zero to find the extreme value of *mse*, as shown in Equation (18).

(18) $$mse=\frac{1}{Q}\sum_{k=1}^{Q}e^{2}\left(k\right)$$

where, *mse* represents the value of the mean square of errors; *Q* represents the total number of samples; *e(k)* represents the error between the actual outputs and the expected outputs.

The second-order partial differential for the square of training error, with respect to the network weight during the *k* cycle, is calculated in Equation (19). The second-order partial differential for the square of training error, with respect to the threshold value during the *k* cycle, is calculated in Equation (20):

(19) $$\frac{\partial e^{2}(k)}{\partial \omega_{ij}}=2e(k)\frac{\partial e(k)}{\partial \omega_{ij}}$$

(20) $$\frac{\partial e^{2}(k)}{\partial b}=2e(k)\frac{\partial e(k)}{\partial b}$$

where *ω_ij_* represents the weight of the network; *b* represents the threshold value of the network.

The first-order partial differential for the square of training error, with respect to the network weight during the *k* cycle is calculated in Equation (21):

(21) $$\frac{\partial e(k)}{\partial \omega_{ij}}=\frac{\partial e}{\partial \omega_{ij}}\{d(k)-[\sum_{i=1}^{R}\omega_{ij}p_{i}(k)+b]\}$$

where *d(k)* represents the expected outputs; *p_i_(k)* represents the inputs. Then, we will obtain Equation (22):

(22) $$\frac{\partial e(k)}{\partial \omega_{ij}}=-p_{i}(k),\;\frac{\partial e(k)}{\partial b}=-1$$

According to the principle of negative gradient descent, the changes of network weights and threshold values should be 2*ηe*(*k*)*p*(*k*) and 2*ηe*(*k*). Therefore, the network weights and threshold values correction Algorithm is shown in Equation (23):

(23) $$\left\{ \begin{array}{l} \omega (k+1)=\omega (k)+2\eta e(k)p^{T}(k)\\ b(k+1)=b(k)+2\eta e(k) \end{array}\right.$$

where *η* represents the learning rate. When the value of *η* is large, the training speed of the network can be accelerated, but if the value of *η* is too large, the stability of the network will be reduced and the training error will be increased. According to previous studies, the value of *η* can be calculated based on the sum of the mean square values for input vectors, as shown in Equation (24).

(24) $$0<\eta <\frac{2}{tr(R)}$$

where *tr(R)* represents the trace of the autocorrelation matrix of the input vector.

**Figure 12:**
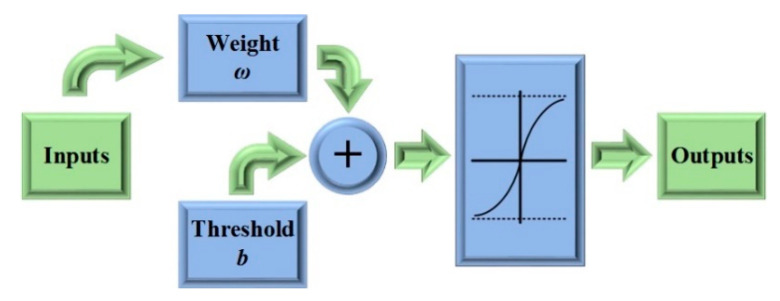
The architecture of the linear neural network.

Then, the linear neural network architecture is shown in Figure 12 and the training steps for the proposed network are as follows:

(1) Define an input variable *p*(*k*), an expected output *d*(*k*), a weight *ω* and a threshold *b*;
(2) Initialize the weight *ω* and threshold *b*;
(3) Input multiple training samples;
(4) Train the network to make *mse* as small as possible through multiple iterations;
(5) Judge whether the algorithm converges or not;
(6) If not, adjust the weight, then recalculate and retrain it.

**Figure 13:**
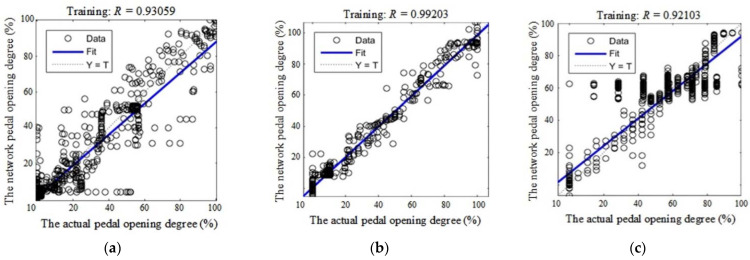
The training results for the linear neural network: (**a**) the result for aggressive driving type; (**b**) the result for general driving type; (**c**) the result for steady driving type.

As mentioned previously, the inputs for the linear neural network are velocity and relative distance, the outputs for the linear neural network are accelerator pedal opening degree or brake pedal opening degree. Thus, through the experimental data with different driving types, the accuracy of the proposed network will be verified by training and testing. The training results are shown in Figure 13.

As shown in Figure 13, the fitted curves for the deviations of the expected pedal opening degree from the actual values are provided with the function *F(T)*. In the Figure 13, the abscissa axis represents the actual values of pedal opening degree and vertical axis represents the expected values of pedal opening degree based on the linear neural network. The dotted lines represent the expected fitted function “*Y = T*”, while the blue lines represent the actual fitted function (*F*(*T*)), which can be calculated in Equation (25):

(25) F(T)≈kT+b

Among them, *k* represents the slope of the fitted curve; *T* represents the actual pedal opening degree; *b* represents the bias of the fitted curve. In this way, if the value of *k* is closer to 1, then it will be better fitting; if the value of *b* is closer to 0, then it will be better for fitting. In addition, *R* is the certainty coefficient, which represents the degree of approximation of the predicted value. The calculation for *R* is shown in Equation (26):

(26) $$R=\sqrt{1-\sum_{i=1}^{l}{\left(y_{i}-{\hat{y}}_{i}\right)}^{2}/\sum_{i=1}^{l}{\left(y_{i}-{\bar{y}}_{i}\right)}^{2}}$$

where $y_{i}$ represents the original data; ${\hat{y}}_{i}$ represents the predicted data; ${\bar{y}}_{i}$ represents the mean of the original data. Based on previous researches, if the value of *R* is closer to 1, then the better the fitting will be. Furthermore, the values of *k* and *b* can be obtained from the training network, which are shown as follows:

$$F(T)=\left\{ \begin{array}{ll} 0.87T+0.014, & \mathrm{aggressive}\\ 0.98T+0.003, & \mathrm{general}\\ 0.85T+0.072, & \mathrm{steady} \end{array}\right.\begin{array}{l} aggressive\\ general\\ steady \end{array}$$

**Figure 14:**
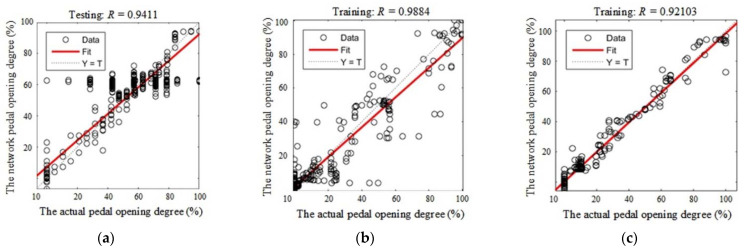
The testing results for the linear neural network: (**a**) the result for aggressive driving type; (**b**) the result for general driving type; (**c**) the result for steady driving type.

After training, we tested each driving type network with random data from the driver samples; the results are shown in Figure 14.

As mentioned previously, in the Figure 14, the abscissa axis represents the actual values of the pedal opening degree and the vertical axis represents the expected values of the pedal opening degree based on the linear neural network. The dotted lines represent the expected fitted function “*Y = T*”, while the red lines represent the actual fitted function (*F*(*T*)), which are the same with the results of training.

**Figure 15:**
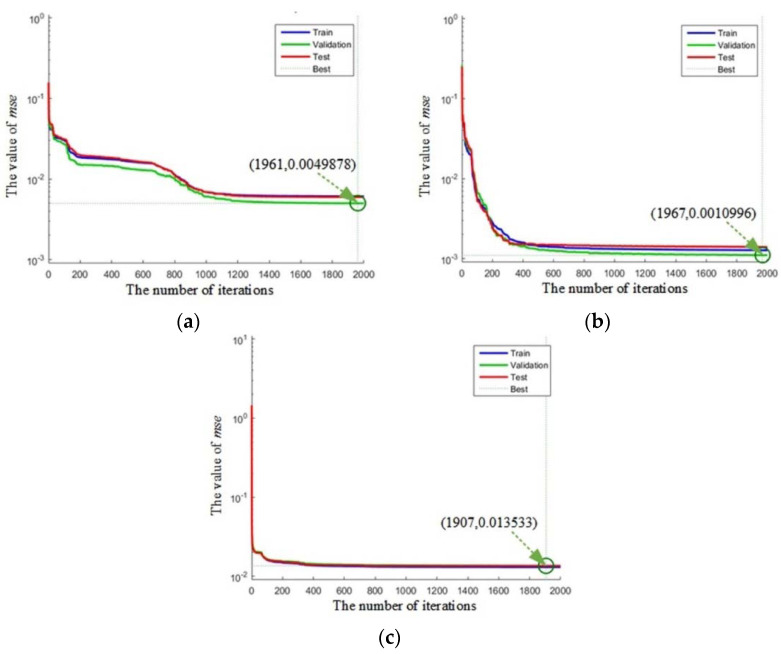
The values of mean square error (*mse*) for the linear neural network: (**a**) the result for aggressive driving type; (**b**) the result for general driving type; (**c**) the result for steady driving type.

Finally, the value of *mse* will approach the best through 2000 iterations; the results are shown in Figure 15.

## 6. Conclusions and Future Works

In daily driving, the driver’s task is to reach the destination safely and efficiently. Thus, different drivers may present different driving behaviors, while their decision-making mechanisms are similar. According to previous studies, the driver will make a certain action based on relative motion states between the target vehicle and the ego vehicle in the field of view. That is to say that the velocity and relative distance jointly determine the driver’s decision-making process. However, the driver’s classification and decision-making were not integrated through conventional studies. Therefore, in order to imitate human-like decision-making during car-following, a brain-inspired decision-making linear neural network was designed to solve this issue. Our study mainly focuses on the following aspects:

(1) The driving simulator was used to collect vehicle state data during car-following with different driver samples.
(2) The proposed fuzzy algorithm can make more precise classifications based on characteristic parameters by extracting and analyzing the experimental data from different driving types.
(3) A brain-inspired linear neural network was established to realize human-like decision-making and control, and its feasibility and accuracy were verified through the training and testing with iteration.

In addition, the sample data size can be increased and the consistency of the output based on the neural network can be improved. Therefore, we will conduct more experiments for different driver samples, and optimize some key parameters in the fuzzy algorithm and neural network in the future.

## 7. Discussions

In this study, some deficiencies and limitations existed. For example, the value of α and β in Equations (16) and (17) were arbitrarily chosen. However, our data collection were based on a driving simulator without the consideration of real vehicle tests.

## Figures and Tables

**Table 1:** The questionnaire for occupant evaluation.

No.	Questions	Answer			Evaluation
1	Push your back when making an acceleration?	Yes	A little	No	Aggressive General Steady
2	Do you nod your head when braking?	Yes	A little	No	
3	Is the speed changing rapidly?	Yes	A little	No	
4	What is the average speed during car-following?	Fast	A little	Slow	
5	What is the relative distance between vehicles during car-following?	Large	A little	small	
6	How about the relative distance between the vehicles after braking?	Large	A little	small	

**Table 2:** An experimental data analysis table of driving behavioral characteristics.

	Male			Female		
	Aggressive	General	Steady	Aggressive	General	Steady
*a_max_* (m/s^2^)	3.9	2.9	2.1	3.6	2.3	1.6
*a_min_* (m/s^2^)	−4.4	−4.4	−2.2	−5.1	−2.6	−2.7
$\bar{a}$ (m/s^2^)	1.5813	1.4917	1.2167	1.5382	1.2061	1.1398
$\sigma_{a}$	2.7891	2.1443	0.7315	2.8296	0.8912	0.5946
*d_safe_* (m)	10	30	60	10	30	60
*d_emergency_* (m)	3.3	4.5	2.4	4.6	2.3	3.2

## Data Availability

Data available in a publicly accessible repository.
